# Diabetic Autonomic Neuropathy and Impaired Inflammatory Reflex

**DOI:** 10.1002/edm2.70349

**Published:** 2026-10-08

**Authors:** Narges Dastmalchi, Mohammad Amin Doustvandi, Reza Rahbarghazi, Khalil Hajiasgharzadeh

**Affiliations:** ^1^ Stem Cell Research Center Tabriz University of Medical Sciences Tabriz Iran; ^2^ Immunology Research Center Tabriz University of Medical Sciences Tabriz Iran

**Keywords:** cholinergic anti‐inflammatory pathway, diabetic autonomic neuropathy, inflammatory reflex, vagus nerve

## Abstract

**Introduction:**

Diabetes mellitus is increasingly recognised as a chronic metabolic‐inflammatory disorder. Diabetic autonomic neuropathy (DAN) is a prevalent yet underdiagnosed complication associated with significant morbidity and mortality. Rather than acting solely as an end‐stage downstream consequence of hyperglycemia, DAN may serve as an active driver of neuroimmune dysfunction by disrupting the inflammatory reflex and its efferent arm, the cholinergic anti‐inflammatory pathway (CAP).

**Methods:**

This review synthesises current preclinical and clinical literature examining the physiological architecture of the inflammatory reflex, the molecular mechanisms linking hyperglycemia‐induced neurovascular damage to CAP failure (including α7 nicotinic acetylcholine receptor (α7nAChR) signalling), and diagnostic modalities (e.g., heart rate variability) used to evaluate autonomic‐immune crosstalk.

**Results:**

Evidence indicates that metabolic stress, oxidative damage, and microvascular ischemia compromise vagal structural integrity and suppress cholinergic anti‐inflammatory signalling. This loss of autonomic restraint exacerbates systemic cytokine production (e.g., TNF‐α, IL‐6), establishing a self‐perpetuating, bidirectional pathophysiological loop that accelerates both neural injury and end‐organ complications. Emerging therapeutic strategies—such as exercise‐induced autonomic reactivation, bioelectronic vagus nerve stimulation (VNS), and targeted α7nAChR agonists—demonstrate promising potential to interrupt this cycle and restore neuroimmune homeostasis.

**Conclusion:**

DAN represents a critical state of autonomic‐mediated immune dysregulation rather than isolated neural damage. While causal mechanisms require further validation through prospective and interventional human trials, reframing DAN as a neuroimmune failure provides novel opportunities for precision phenotyping and targeted therapeutic interventions.

## Graphical abstract

The cholinergic anti‐inflammatory pathway under normal and diabetic conditions. In the normal state, vagal activation through nicotinic acetylcholine receptors helps regulate immune responses and suppress excessive inflammation. In diabetes, impaired autonomic function and dysregulated cholinergic signalling weaken this anti‐inflammatory pathway, contributing to persistent inflammation, immune dysfunction and disease progression.

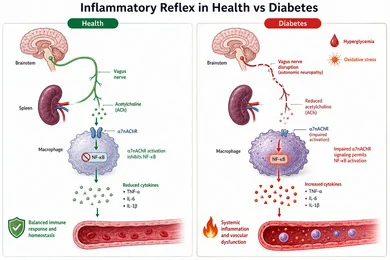

## 1 Introduction

Diabetes mellitus is traditionally defined by abnormalities in glucose metabolism; however, it is increasingly recognised as a chronic, multisystem disorder characterised by persistent metabolic stress, oxidative injury, microvascular dysfunction and low‐grade inflammation [1, 2]. Despite advances in glycemic management, diabetic complications remain a leading cause of morbidity and mortality worldwide [3]. Among these, diabetic autonomic neuropathy (DAN) is particularly concerning, as it is strongly associated with cardiovascular mortality, impaired quality of life, and progressive end‐organ dysfunction [4].

Current management strategies for DAN are largely preventive or symptomatic [5]. Yet, growing evidence suggests that autonomic dysfunction is not merely a downstream complication. Instead, it may actively drive neuroimmune and inflammatory dysregulation, contributing to the broader systemic alterations and metabolic decline seen in diabetes [6, 7]. These observations support the hypothesis that DAN could represent a potentially modifiable neuroimmune lesion that perpetuates inflammatory activation and accelerates the progression of diabetic complications.

This conceptual framework posits that DAN compromises the integrity of the inflammatory reflex. This impairment fosters a chronic pro‐inflammatory milieu, which in turn facilitates the progression of diabetic complications [8]. This hypothesis is particularly relevant given the early onset of parasympathetic withdrawal in DAN, which has been most consistently documented in patients with subclinical or early‐stage DAN and is often identified through reduced heart rate variability (HRV) in individuals with diabetes before overt autonomic symptoms become clinically apparent [9, 10]. This withdrawal occurs alongside the sustained inflammatory activation characteristic of diabetes. Such observations suggest a bidirectional pathophysiological loop. In this cycle, systemic inflammation exacerbates neural injury, while autonomic failure simultaneously diminishes the neural restraint of inflammatory responses, resulting in a self‐reinforcing process of neuroimmune dysregulation.

In this review, we examine the hypothesis that DAN represents a clinical model of impaired neuroimmune homeostasis. We summarise the clinical spectrum and pathogenesis of DAN, review the physiological basis of the inflammatory reflex and cholinergic anti‐inflammatory pathway (CAP), and discuss how diabetes‐related autonomic dysfunction may weaken anti‐inflammatory neural control. Finally, we consider therapeutic implications and future research directions.

## 2 Diabetic Autonomic Neuropathy (DAN): Clinical Spectrum and Pathophysiology

DAN is a serious and often underrecognized complication of diabetes mellitus characterised by damage to autonomic nerve fibres that innervate multiple organ systems [11]. It may involve both sympathetic and parasympathetic pathways and can affect cardiovascular, gastrointestinal, genitourinary and sudomotor functions [12, 13]. DAN is associated with significant morbidity, impaired quality of life, and increased mortality, particularly when cardiovascular autonomic involvement is present [14]. The condition may develop in both Type 1 and Type 2 diabetes and is closely related to disease duration, metabolic control, and the presence of other microvascular complications [15, 16, 17].

Among the various manifestations of DAN, cardiac autonomic neuropathy (CAN) is the most extensively studied and clinically relevant [18, 19]. CAN impairs cardiovascular control, manifesting as resting tachycardia, exercise intolerance, reduced HRV, baroreflex dysfunction, and orthostatic hypotension, while increasing the risk of arrhythmias and silent myocardial ischemia [20, 21]. Importantly, parasympathetic dysfunction often appears earlier than sympathetic impairment, and reductions in HRV are commonly considered one of the earliest detectable markers of CAN [22, 23]. Because vagal activity plays a central role in autonomic regulation, early parasympathetic dysfunction may have broader physiological implications beyond cardiovascular control [24].

The pathogenesis of DAN is multifactorial and reflects the complex metabolic and vascular disturbances associated with diabetes [25]. Chronic hyperglycemia initiates several biochemical pathways that contribute to neuronal injury, including activation of the polyol pathway, accumulation of advanced glycation end‐products (AGEs), activation of protein kinase C, and increased flux through the hexosamine pathway [26, 27, 28]. These metabolic disturbances promote oxidative and nitrosative stress, mitochondrial dysfunction, and abnormal intracellular signalling, ultimately leading to structural and functional damage to autonomic neurons [29, 30].

Inflammatory processes have also been increasingly implicated in the pathogenesis of diabetic neuropathies [31, 32]. Diabetes is associated with chronic low‐grade systemic inflammation characterised by elevated levels of pro‐inflammatory mediators such as tumour necrosis factor‐α (TNF‐α), interleukin‐6 (IL‐6) and C‐reactive protein [33, 34]. These inflammatory mediators may contribute to neuronal damage through oxidative stress, endothelial dysfunction and direct neurotoxic effects [35, 36, 37, 38]. Furthermore, inflammatory signalling may interact with metabolic and vascular pathways, amplifying neural injury and accelerating the progression of autonomic dysfunction [39].

Taken together, DAN arises from the interplay of metabolic dysregulation, oxidative stress, microvascular injury and inflammatory activation. These mechanisms contribute to progressive damage of autonomic nerve fibres and disruption of autonomic control across multiple organ systems. Given the central role of autonomic pathways in maintaining physiological homeostasis, dysfunction of these neural circuits may have consequences that extend beyond traditional autonomic symptoms, potentially influencing broader regulatory processes including neuroimmune interactions.

## 3 The Inflammatory Reflex and the CAP

The nervous and immune systems interact closely to maintain physiological homeostasis during infection, injury and metabolic stress [40]. One of the key mechanisms underlying this interaction is the inflammatory reflex, a neuroimmune regulatory circuit through which the nervous system detects inflammatory signals and modulates immune responses [24, 41, 42]. This reflex mechanism functions analogously to other homeostatic reflexes, involving afferent sensing of inflammatory stimuli, central integration within the central nervous system, and efferent autonomic pathways that regulate immune activity [43, 44, 45]. The afferent arm of this reflex has been most robustly demonstrated in preclinical animal studies, whereas evidence in humans is more limited and is derived primarily from physiological, clinical and translational observations. Through this coordinated neural control, the inflammatory reflex helps prevent excessive or uncontrolled cytokine production that could otherwise lead to tissue injury and systemic inflammation.

Peripheral inflammatory signals, including cytokines, pathogen‐associated molecular patterns, and damage‐associated molecular patterns, can activate sensory pathways that convey information to central autonomic regulatory centres [24]. Afferent fibres of the vagus nerve are believed to play a central role in this process by transmitting signals from peripheral tissues to the brainstem, where inflammatory information is integrated with other autonomic inputs [42]. These signals are processed primarily in nuclei within the medulla, including the nucleus tractus solitarius, which coordinates autonomic responses and relays information to higher regulatory centres [46].

The efferent arm of the inflammatory reflex involves autonomic pathways that regulate immune cell activity and cytokine production [47]. Among these mechanisms, the CAP represents a key neural mechanism for limiting inflammation [48, 49]. In this pathway, acetylcholine‐mediated signalling inhibits the release of pro‐inflammatory cytokines from immune cells, thereby providing a neural brake on inflammatory responses [50]. Experimental studies have demonstrated that cholinergic signalling can suppress the production of several important inflammatory mediators, including TNF‐α, IL‐6, IL‐1β [51, 52, 53].

A central component of the CAP is the interaction between acetylcholine and nicotinic acetylcholine receptors expressed on immune cells [54, 55]. In particular, the α7 nicotinic acetylcholine receptor (α7nAChR) expressed on macrophages and other immune cells has been identified as an important mediator of cholinergic immunomodulation [56, 57]. Activation of these receptors has been shown to inhibit nuclear factor‐κB signalling pathways and reduce cytokine synthesis, thereby limiting the magnitude of inflammatory responses [58, 59, 60, 61]. Although the precise anatomical and cellular intermediates of this pathway remain an area of active investigation [62], the functional role of cholinergic signalling in regulating immune responses is well supported by experimental and translational studies.

The inflammatory reflex, mediated by vagal cholinergic signalling, serves as a critical mechanism for restraining systemic inflammation, with HRV acting as a key clinical surrogate for vagal efficacy [63]. In diabetes, autonomic impairment associated with DAN weakens this neural restraint, thereby promoting a pro‐inflammatory milieu. Consequently, the loss of cholinergic tone likely exacerbates sustained cytokine activation, positioning this autonomic neuropathy as a potential mechanistic driver of chronic inflammation and progressive metabolic complications. However, it is important to emphasise that this model is primarily based on current experimental and observational evidence; definitive confirmation of causality will require further longitudinal and interventional studies to validate this framework in clinical populations.

## 4 Linking DAN to Impaired Inflammatory Reflex

Emerging evidence suggests that DAN may disrupt neuroimmune regulation, particularly mechanisms underlying the inflammatory reflex [64]. Structural and functional impairment of vagal pathways in diabetes could reduce neural restraint on cytokine production [65] (Figure 1). Consistent with this possibility, reductions in HRV, which is a clinical surrogate of vagal tone, have been repeatedly associated with elevated inflammatory markers, including C‐reactive protein, TNF‐α and IL‐6 [8, 66]. In addition to vagal dysfunction, diabetes‐related alterations in immune cell responsiveness may further compromise the CAP [67, 68]. Experimental data indicate that hyperglycemia and oxidative stress can modify the expression or functional sensitivity of macrophage nicotinic acetylcholine receptors, including the α7nAChR, potentially reducing cholinergic suppression of cytokine release [69, 70].

**FIGURE 1:**
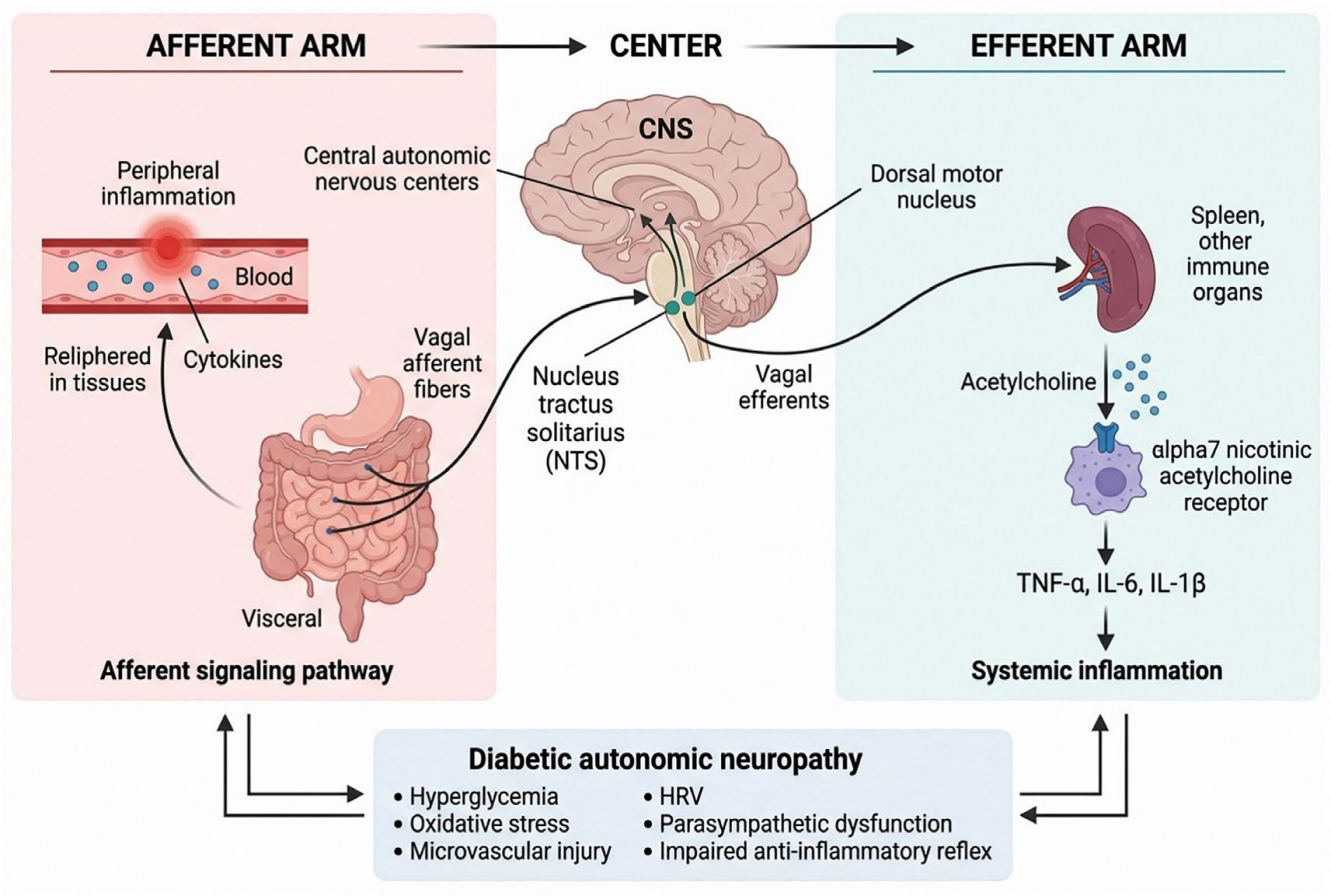
Schematic representation of the cholinergic anti‐inflammatory pathway and its impairment in diabetic autonomic neuropathy. The afferent arm (left) detects peripheral inflammation and transmits signals via vagal afferents to the nucleus tractus solitarius (NTS). The central autonomic centres integrate this signal, engaging the efferent arm (right), where vagal outflow from the dorsal motor nucleus leads to acetylcholine (ACh) release. ACh binding to α7 nicotinic acetylcholine receptors (α7nAChR) on macrophages inhibits the production of pro‐inflammatory cytokines (TNF‐α, IL‐6, IL‐1β). The lower panel summarizes how diabetic autonomic neuropathy—driven by hyperglycaemia, oxidative stress and microvascular injury—induces parasympathetic dysfunction and reduced heart rate variability (HRV), thereby compromising the anti‐inflammatory reflex and exacerbating systemic inflammation.

Conversely, inflammatory mediators may contribute to neuronal injury through oxidative stress, endothelial dysfunction and microvascular damage [71]. This reciprocal interaction raises the possibility of a reinforcing cycle in which autonomic injury and inflammation amplify one another [72]. Although the temporal and causal relationships remain incompletely defined, this framework suggests that DAN may contribute to impaired inflammatory reflex activity and sustained inflammatory activation in diabetes. The proposed neuroimmune interaction is illustrated in Figure 2.

**FIGURE 2:**
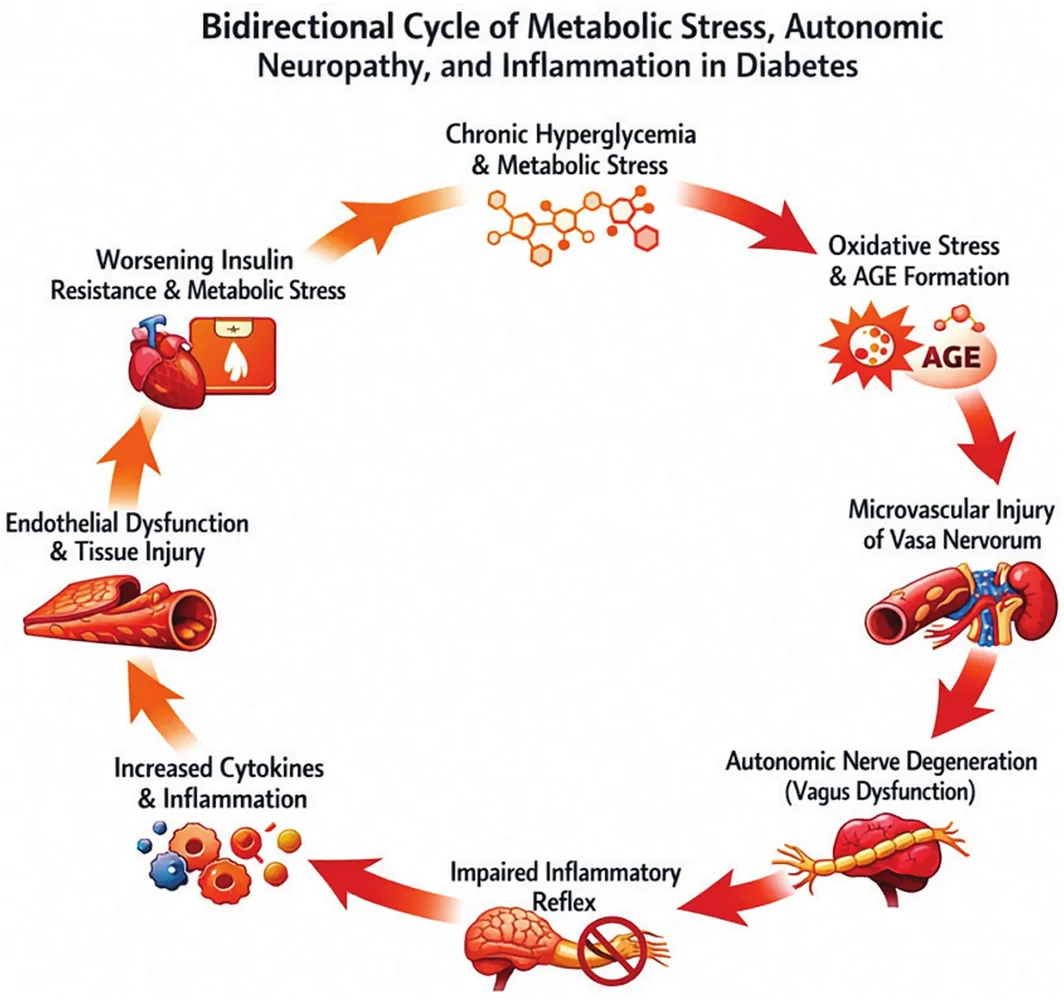
Bidirectional vicious cycle linking metabolic stress, autonomic injury, and systemic inflammation in diabetes. Chronic hyperglycemia, oxidative stress, and the accumulation of advanced glycation end products (AGEs) drive low‐grade inflammation and microvascular dysfunction, establishing an injurious metabolic environment. Concurrently, ongoing inflammatory signalling and microvascular injury exacerbate autonomic fibre damage, worsening vagal dysfunction and weakening cholinergic anti‐inflammatory restraint. This loss of autonomic buffering amplifies immune activation, reinforcing a self‐perpetuating cycle of metabolic injury and neuroimmune dysregulation. This conceptual model illustrates a plausible bidirectional interaction that requires further mechanistic validation.

## 5 Mechanisms by Which Diabetes May Disrupt the CAP

The disruption of the CAP in diabetes is likely not the result of a single lesion but rather a consequence of a complex, multifactorial pathophysiology. This impairment emerges from the intersection of structural neuro‐axonal degeneration, metabolic‐vascular insults and altered immune cell sensitivity. Together, these elements create a milieu that progressively undermines the capacity of the vagus nerve to restrain systemic inflammation.

### 5.1 Structural and Functional Vagal Insufficiency

The fundamental integrity of the inflammatory reflex relies on the anatomical and functional continuity of the vagus nerve [73, 74]. In the diabetic state, chronic hyperglycemia and the activation of accessory metabolic pathways—specifically the polyol and hexosamine pathways, along with the accumulation of AGEs—exert direct neurotoxic effects [75]. As a result, autonomic fibres, particularly the parasympathetic fibres of the vagus nerve, are highly susceptible to metabolic stress [76]. This leads to structural rarefaction, demyelination and axonal degeneration.

Beyond structural loss, functional transmission is compromised. The metabolic environment of diabetes impairs neurotransmitter synthesis, release, and synaptic transmission efficiency at both the ganglionic and terminal levels [77]. Consequently, even in early‐stage DAN, where explicit neuropathy may not yet be clinically apparent, the threshold for vagal activation is elevated, and the magnitude of acetylcholine release at peripheral sites is diminished, creating a deficit in the primary efferent signal required for anti‐inflammatory signalling.

### 5.2 Microvascular Compromise and Ischemic Injury

Microvascular dysfunction, a hallmark of diabetes, is intimately linked to autonomic neural degeneration [78]. Structural remodelling of the vasa nervorum, which is characterised by basement membrane thickening and endothelial dysfunction, limits the perfusion and oxygenation of autonomic nerves [79]. This ischemia–reperfusion injury exacerbates oxidative stress, accelerating the degradation of nerve fibres. As the functional density of vagal efferents decreases, the capacity for efferent vagal signalling to reach target immune organs (such as the spleen and liver) is impaired, effectively disconnecting the central nervous system from its pivotal roles in immune responses management.

### 5.3 Immunometabolic Priming and Receptor‐Level Dysfunction

The efficacy of the CAP is also dependent on the sensitivity of target immune cells to acetylcholine. In diabetes, the chronic pro‐inflammatory environment induces ‘priming’ of macrophages and monocytes toward a pro‐inflammatory phenotype [80]. In the setting of reduced vagal tone, this immunometabolic priming may be insufficiently counterbalanced by cholinergic inhibitory input [81]. Thus, diminished autonomic restraint could amplify the inflammatory bias already present in diabetes, potentially magnifying cytokine output beyond what metabolic factors alone would generate.

Furthermore, the α7nAChR, the principal mediator of cholinergic cytokine inhibition, is sensitive to the surrounding biochemical environment [82]. Oxidative stress, which is rampant in the diabetic milieu, can induce post‐translational modifications or conformational changes in the α7nAChR complex, potentially decreasing receptor affinity or internal signalling efficacy [83]. While human evidence regarding α7nAChR sensitivity in diabetes is currently scarce, these mechanistic pathways suggest that the anti‐inflammatory signals transmitted by the vagus nerve are often received by unresponsive immune cells (Table 1).

**TABLE 1:** The potential mechanisms linking diabetic autonomic neuropathy (DAN) to impaired inflammatory reflex.

Mechanistic domain	Pathophysiological process	Consequence for inflammatory reflex	Key mediators/indicators	References
Metabolic injury	Chronic hyperglycemia activates polyol pathway, AGE accumulation, protein kinase C signalling and hexosamine flux	Direct neuronal toxicity and autonomic fibre degeneration	AGEs, sorbitol accumulation, PKC activation	[26, 27, 28]
Oxidative stress	Hyperglycemia‐driven mitochondrial dysfunction increases ROS and nitrosative stress	Damage to autonomic neurons and impaired neurotransmission	ROS, mitochondrial dysfunction	[30]
Neuroinflammation	Diabetes‐associated systemic inflammation exposes nerves to pro‐inflammatory cytokines	Cytokine‐mediated neuronal injury and autonomic dysfunction	TNF‐α, IL‐6, CRP	[33, 34]
Vagal dysfunction	Degeneration of parasympathetic fibres reduces vagal efferent activity	Loss of neural restraint on cytokine production via CAP	Reduced HRV, vagal tone decline	[8, 22, 63]
Microvascular injury	Structural damage to the vasa nervorum limits perfusion of autonomic nerves	Ischemia‐induced degeneration of vagal fibres	Endothelial dysfunction, basement membrane thickening	[78, 79]
Receptor‐level immune dysfunction	Hyperglycemia and oxidative stress alter macrophage receptor sensitivity	Reduced cholinergic suppression of cytokine production	α7nAChR dysfunction	[69, 83]
Immunometabolic priming	Chronic metabolic inflammation biases immune cells toward a pro‐inflammatory phenotype	Amplified cytokine release despite reduced vagal modulation	Activated macrophages, monocytes	[80]
Neuroimmune feedback loop	Autonomic dysfunction increases inflammation, which further damages nerves	Self‐reinforcing cycle of neuroimmune dysregulation	Reduced HRV + elevated cytokines	[72]

## 6 Assessment of DAN in the Context of Inflammation

To evaluate the hypothesis that DAN impairs the inflammatory reflex, clinical assessment should move beyond the identification of isolated organ dysfunction and instead focus on the integrity of the neuroimmune axis [84]. A comprehensive framework requires integration of physiological autonomic testing with measurement of systemic inflammatory biomarkers, thereby capturing both autonomic regulation and immune activation. Standard cardiovascular autonomic reflex tests remain the clinical reference for the diagnosis of CAN [85]. These assessments, including heart rate responses to deep breathing, the Valsalva manoeuvre, and orthostatic challenge, provide validated measures of efferent autonomic function [84]. In parallel, HRV analysis offers a noninvasive index of vagal tone and autonomic balance [86, 87, 88].

To determine whether autonomic impairment is accompanied by dysregulated immune control, autonomic testing should be complemented by assessment of circulating inflammatory markers [89]. In particular, measurement of pro‐inflammatory cytokines such as TNF‐α, IL‐6, and IL‐1β may provide a useful, although indirect, readout of CAP function [41, 90]. The relationship between reduced HRV and elevated cytokine levels may therefore serve as an initial indicator of neuroimmune dissociation. At present, direct evaluation of inflammatory reflex integrity remains largely confined to the research setting. Parameters such as cholinergic signalling capacity, α7nAChR function, and vagal efferent activity are not routinely incorporated into clinical practice. Accordingly, while a combined neuroimmune profiling approach is conceptually attractive, its translation into standard care will require further methodological refinement and clinical validation.

The longer‐term goal is the development of a stratified neuroimmune phenotype in diabetes. Such an approach would integrate autonomic indices, inflammatory biomarkers, and clinical complication profiles to identify patients with distinct patterns of neuroimmune dysfunction. Mapping clusters of complications, such as CAN, nephropathy, and gastroparesis, against quantitative measures of autonomic impairment and inflammatory burden may improve risk stratification and facilitate recognition of individuals who could benefit from targeted interventions, including neuromodulatory or anti‐inflammatory strategies aimed at restoring autonomic and immune homeostasis.

## 7 Therapeutic Implications

Therapeutic management of DAN must evolve from standard symptomatic relief toward the restoration of neuroimmune homeostasis. Addressing DAN effectively requires a multi‐pronged approach that bridges foundational metabolic stabilisation with emerging strategies aimed at direct modulation of the inflammatory reflex.

### 7.1 Metabolic and Pharmacologic Stabilisation

Strict glycemic control remains the cornerstone for preventing further neuro‐axonal degeneration [91]. By mitigating hyperglycemia and oxidative stress, metabolic stabilisation reduces the systemic pro‐inflammatory milieu that drives autonomic injury [92]. Concurrently, pharmacological management is increasingly leveraging agents with pleiotropic effects—such as those enhancing nitric oxide bioavailability or microvascular perfusion—that extend beyond glucose lowering [93]. Emerging evidence suggests that specific anti‐diabetic drug classes, particularly Glucagon‐like peptide‐1 receptor agonists (GLP‐1RAs) and Sodium‐glucose cotransporter‐2 (SGLT2) inhibitors, provide significant neuro‐ and cardio‐protective benefits by modulating inflammatory signalling and enhancing autonomic tone [94, 95, 96]. These agents have been shown to attenuate systemic inflammation, reduce oxidative stress, and improve HRV, thereby serving as critical adjuncts to support nerve health and improve autonomic signal transmission (Table 2). Such precision management may halt the progression of autonomic dysfunction by directly targeting the metabolic and inflammatory substrates of DAN.

**TABLE 2:** Pleiotropic effects of anti‐diabetic agents on autonomic and inflammatory function.

Pharmacologic class	Primary mechanism	Pleiotropic effects	Relevance to autonomic function	References
GLP‐1 receptor agonists	GLP‐1 receptor activation	Anti‐inflammatory, antioxidant, improved endothelial function	Enhanced baroreflex sensitivity; improved HRV	[97, 98]
SGLT2 inhibitors	Inhibition of glucose reabsorption	Reduced systemic inflammation, improved metabolic energy substrate utilisation	Reduced sympathetic overactivity; cardioprotective remodelling	[99, 100, 101]
Metformin	AMPK activation	Anti‐inflammatory, improved microvascular circulation	Mitigation of oxidative stress‐induced nerve injury	[102, 103]

### 7.2 Autonomic Reactivation and Neuromodulation

Strategies to restore vagal efferent activity are central to the new neuroimmune‐focused management paradigm. Lifestyle modifications, particularly regular physical activity, provide consistent physiological stimuli that enhance HRV and endogenous anti‐inflammatory capacity. In patients with advanced autonomic dysfunction, investigational neuromodulatory approaches may help modulate residual neuroimmune signalling despite underlying structural nerve injury [6, 104]. Targeted neuromodulation, such as vagus nerve stimulation (VNS), and pharmacological agonists of the α7nAChR, may allow for direct manipulation of the inflammatory reflex. However, it is important to note that the application of these strategies specifically for diabetic autonomic neuropathy remains largely experimental, and clinical evidence supporting their efficacy in this population is currently limited. Despite these limitations, these precision‐medicine approaches represent a significant shift toward restoring the body's innate capacity to resolve inflammation, offering a potential pathway to improve clinical outcomes in patients with advanced diabetic complications.

## 8 Future Directions

The integration of neuroimmune dysregulation into the pathogenesis of DAN represents a burgeoning area of inquiry. However, several critical knowledge gaps must be addressed to transition from conceptual frameworks to clinical application.

### 8.1 Establishing Causality Versus Association

Currently, the body of human evidence linking autonomic dysfunction with systemic inflammation is largely observational and correlational. It remains difficult to determine whether impaired autonomic regulation is a contributory factor of sustained inflammation, or if both phenomena occur in parallel as independent manifestations of systemic metabolic injury. Future longitudinal investigations are essential to delineate the temporal relationships between the decline of vagal tone and the escalation of inflammatory markers. Such studies should employ dynamic modelling to determine whether autonomic degradation precedes or is subsequently exacerbated by the progression of systemic inflammation [24, 105].

### 8.2 Refining Metrics of the Inflammatory Reflex

While HRV serves as an accessible surrogate for vagal tone, its limitations as a proxy for inflammatory reflex integrity are significant [106, 107]. HRV is influenced by a multitude of non‐neurological confounders, and it does not capture the specific efferent signalling capacity of the CAP. Future research should prioritize the development and validation of integrated neuroimmune biomarkers. This may involve combining spectral analysis of HRV with direct measurements of vagal efferent activity, where feasible, or utilising novel biomarkers that reflect the specific downstream activation of the cholinergic pathway, such as phosphorylated transcription factors downstream of the α7nAChR receptor [73].

### 8.3 Accounting for Disease Heterogeneity

The pathogenesis, inflammatory landscape, and autonomic profiles of diabetes are not uniform. Distinctions between Type 1 and Type 2 diabetes—including differences in age of onset, obesity, insulin resistance, and chronicity—likely influence the neuroimmune phenotype [108, 109]. Furthermore, the confounding effects of co‐morbidities such as hypertension, chronic kidney disease, and the pharmacological impact of common medications (e.g., beta‐blockers, ACE inhibitors) must be rigorously controlled. Future clinical studies should implement stratified analyses to account for these variables, ensuring that findings regarding neuroimmune dysfunction are robust across diverse patient cohorts.

### 8.4 Mechanistic Validation in Human Models

There is a pressing need for human translational studies to move beyond systemic biomarker associations toward mechanistic validation. Specifically, research should investigate whether the chronic diabetic milieu induces cellular desensitisation to cholinergic input. Characterising the expression, conformational state, and intracellular signalling efficacy of the α7nAChR on peripheral blood mononuclear cells in diabetic patients compared to age‐matched controls is a vital next step. Demonstrating that diabetes‐associated oxidative stress effectively creates ‘cholinergic resistance’ at the cellular level would provide a definitive mechanistic basis for the proposed neuroimmune disconnect.

### 8.5 Interventional Trials

Ultimately, the validity of the neuroimmune hypothesis in diabetes will be determined by interventional success. The field requires randomised controlled trials designed to assess whether targeted restoration of autonomic function—whether through intensive lifestyle modification, neuromodulation (e.g., auricular VNS), or pharmacologic enhancement of the cholinergic pathway—results in a meaningful reduction in systemic inflammatory burden and, by extension, a mitigation of diabetic microvascular complications. Such trials will be essential to determine whether the ‘inflammatory reflex’ is a modifiable therapeutic target in the clinical management of diabetes.

## 9 Conclusion

In conclusion, DAN represents a significant and underappreciated systemic failure of neuroimmune homeostasis. Beyond its classic presentation as a collection of organ‐specific deficits, the loss of vagal tone and the subsequent impairment of the inflammatory reflex constitute a fundamental disruption of the body's innate capacity to resolve systemic inflammation. This review proposes that DAN may function not merely as a downstream complication of chronic hyperglycemia, but potentially as a bidirectional contributor to systemic morbidity.

The recognition of this neuro‐immune dissociation offers a refined paradigm for future clinical research and patient care. By reframing DAN as a state of autonomic‐mediated immune dysregulation, we gain a more comprehensive understanding of the pathogenesis of diabetic microvascular disease. This conceptual shift suggests that current therapeutic strategies, which prioritize glycemic regulation, may be necessary but insufficient: optimal management must eventually address the restoration of the CAP. While this mechanism has been demonstrated in animal models of diabetes, validation in human patient cohorts remains a critical knowledge gap.

Ultimately, the convergence of diabetology, neurology, and immunology represented by this framework opens new frontiers for precision medicine. Whether through targeted neuromodulation, structured lifes tyle‐based autonomic reactivation, or novel immunopharmacology, the objective of restoring the inflammatory reflex provides a compelling strategy to mitigate the long‐term burden of diabetes. As translational research in this domain progresses, the validation of this neuro‐immune model promises to fundamentally shift our approach, moving from the management of symptomatic decline toward the proactive preservation of autonomic and inflammatory balance in patients living with diabetes.

## Author Contributions

**Narges Dastmalchi:** conceptualization, writing – original draft. **Mohammad Amin Doustvandi:** conceptualization, writing – review and editing. **Reza Rahbarghazi:** conceptualization, writing – review and editing, methodology, supervision. **Khalil Hajiasgharzadeh:** conceptualization, methodology, validation, writing – review and editing, funding acquisition, supervision.

## Funding

The grant for this study was provided by the National Institute for Medical Research Development (4030695).

## Ethics Statement

The authors have nothing to report.

## Consent

The authors have nothing to report.

## Conflicts of Interest

The authors declare no conflicts of interest.

## Data Availability

Data sharing not applicable to this article as no datasets were generated or analysed during the current study.
